# Bioactive inorganic particles‐based biomaterials for skin tissue engineering

**DOI:** 10.1002/EXP.20210083

**Published:** 2022-03-17

**Authors:** Jingge Ma, Chengtie Wu

**Affiliations:** ^1^ State Key Laboratory of High Performance Ceramics and Superfine Microstructure Shanghai Institute of Ceramics Chinese Academy of Sciences Shanghai P. R. China; ^2^ Center of Materials Science and Optoelectronics Engineering University of Chinese Academy of Sciences Beijing P. R. China

**Keywords:** inorganic biomaterial, nanoparticle, tissue engineering, wound healing

## Abstract

The challenge for treatment of severe cutaneous wound poses an urgent clinical need for the development of biomaterials to promote skin regeneration. In the past few decades, introduction of inorganic components into material system has become a promising strategy for improving performances of biomaterials in the process of tissue repair. In this review, we provide a current overview of the development of bioactive inorganic particles‐based biomaterials used for skin tissue engineering. We highlight the three stages in the evolution of the bioactive inorganic biomaterials applied to wound management, including single inorganic materials, inorganic/organic composite materials, and inorganic particles‐based cell‐encapsulated living systems. At every stage, the primary types of bioactive inorganic biomaterials are described, followed by citation of the related representative studies completed in recent years. Then we offer a brief exposition of typical approaches to construct the composite material systems with incorporation of inorganic components for wound healing. Finally, the conclusions and future directions are suggested for the development of novel bioactive inorganic particles‐based biomaterials in the field of skin regeneration.

## Graphical abstract

The inorganic biomaterials for wound healing have aroused widespread concern in the field of tissue engineering. The three main forms of application of inorganic particles for skin repair are summarized based on the complexity of material system. The representative types of inorganic biomaterials and fabrication technology of wound dressings are presented.

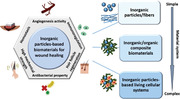

## 1 INTRODUCTION

As the largest organ in human body, skin protects the internal environment from the harmful invasion of mechanical, chemical, and biological stressors.^[^ ^1^ ^]^ Conventionally, adult skin is generally described as consisting of two layers: non‐vascularized epidermis and underlying dermis. The dermis incorporates vascular, neural, and lymphatic systems, as well as multiple appendages such as secretary glands, hair follicles, and nails.^[^ ^2^ ^]^ With a multilayered structure, the skin possesses various biological functions including immune‐competence, endocrine, psycho‐emotion, ultraviolet radiation sensing to maintain temperature, electrolyte, and fluid balance of the mammalian body.^[^ ^1^, ^3^ ^]^

However, deep lacerations, severe burns, surgical operations, pressure, venous stasis, diabetes mellitus, and genetic disorders can destroy the integrity and functionality of skin tissue and result in cutaneous damages.^[^ ^2^, ^3^, ^4^ ^]^ Usually the cutaneous wounds are classified into two main types: acute wounds and chronic wounds on the basis of repair process.^[^ ^5^ ^]^ Acute wounds may arise from mechanical and thermal injuries (accidents, burns, surgery, chemical corrosion, etc.) with a tendency to heal rapidly and completely. After damage, cutaneous wound healing occurs via a series of overlapping dynamic process, which associates with multiple cell types, tissues, biomolecules, growth factors, and enzymes.^[^ ^5a^ ^]^ The acute wound healing process in human body involves several crucial events: (a) rapid hemostasis; (b) appropriate inflammation; (c) migration, proliferation, and differentiation of multiple skin cells; (d) sufficient angiogenesis; (e) re‐epithelialization; and (f) deposition and alignment of collagen.^[^ ^6^ ^]^ Nevertheless, intrinsic pathobiological abnormalities and extrinsic unfavorable factors, including infection, thrombosis, and ischaemia, may lead to complex microenvironment in wound bed.^[^ ^4a^ ^]^ In this instance, chronic wound occurs. The chronic wounds hardly tend to rapid concrescence and always follow an unresponsive and irregular progress of wound healing.^[^ ^4^, ^7^ ^]^ In addition, cutaneous wounds can also be classified based on the depth of the skin tissue affected. The injury involving epidermis only is called superficial wound, and the injury that affects both epidermis and dermis is known as partial thickness wound.^[^ ^5b^ ^]^ More seriously, full‐thickness wound refers to the injury that damages deeper subcutaneous tissue.

In fact, it is difficult for dermis of adult mammals to regenerate spontaneously in the full‐thickness or chronic wound beds, and epithelization only occurs on the premise of the existence of dermal matrix.^[^ ^8^ ^]^ At present, autotransplantation has become the best clinic option for cutaneous damages, but it is restricted by the shortage of autologous donor.^[^ ^9^ ^]^ Moreover, the application of allografts and xenografts is at risk of bacterial infection and immune rejection.^[^ ^10^ ^]^

To address the issues above, skin tissue engineering has aroused wide concern during the past few decades. The ultimate goal of skin tissue engineering is to guide the growth of a living organ that can offer complete biofunctions to replace damaged or diseased skin permanently.^[^ ^1^, ^11^ ^]^ From this perspective, the innovative materials with multi‐biofunctions are urgently needed in the field of skin tissue engineering. Through combining biomaterials, cells, and bioactive molecules, artificial skin substitutes hold great promise to be applied in the treatment of cutaneous damages to promote skin regeneration. Basically, an ideal tissue engineered skin substitute should prevent water loss and microbial contamination, deliver bioactive components, support cell migration, proliferation, and differentiation,^[^ ^12^ ^]^ so that various biomaterials with high biological activities are required to deal with different cutaneous wounds. Recently, bioactive inorganic materials exhibit great potential in enhancing soft tissue repair, hence researchers have designed multi‐functional inorganic particles‐based biomaterials to improve skin regeneration therapies.^[^ ^13^ ^]^ Ninan et al. concerned bio‐composite scaffolds based on polymer/inorganic material for wound healing.^[^ ^14^ ^]^ They highlighted the great potential of inorganic materials in skin regeneration. Besides, Kargozar et al. provided detailed explanation of the usage of hard bioceramics in soft tissue regeneration.^[^ ^15^ ^]^ They discussed the interactions between bioceramics‐containing systems and soft tissue and affirmed the actual suitability of inorganic materials in soft tissue regeneration. Therefore, the inorganic particle‐based biomaterials have great opportunity to become competitive candidates for efficient skin regeneration considering their desirable properties such as hemostasis, angiogenesis, immunoregulation, antibacterial property, promoting hair follicle regeneration, enhanced mechanical strength, and so on.

**FIGURE 1:**
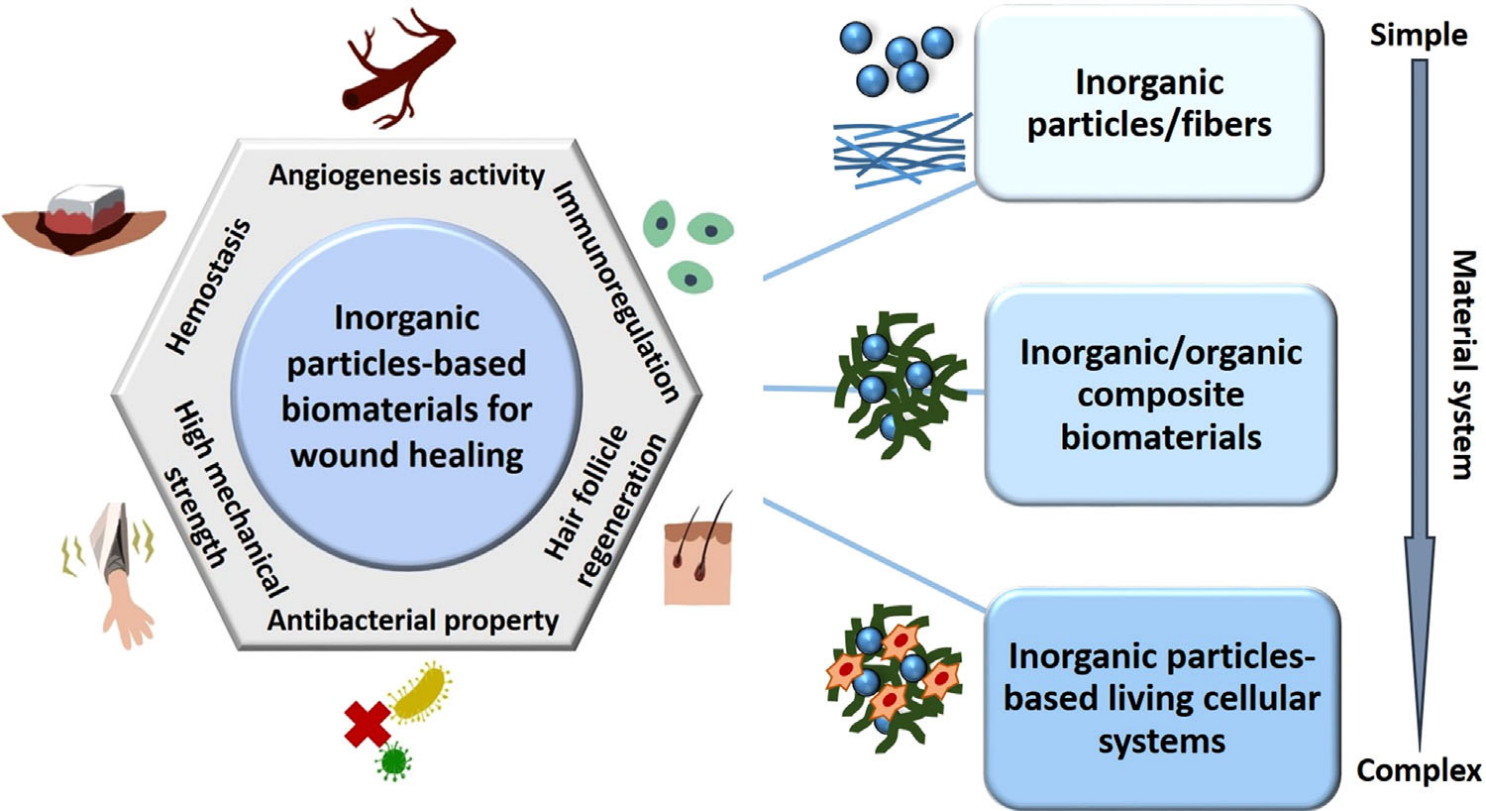
Schematic illustration of the three types of inorganic particles‐based biomaterials classified by complexity of the material system for various applications of wound healing

In this review article, we critically focus on current inorganic particles‐based biomaterials with various biofunctions for wound repair and skin regeneration. We summarize the three main application forms of inorganic biomaterials in the field of skin tissue engineering, including inorganic particles/fibers, inorganic/organic composite scaffolds, and living cellular systems containing inorganic particles (Figure 1). We first describe the types of inorganic biomaterials that can treat skin wound directly. Subsequently, we highlight multiple inorganic/organic composite biomaterials for promoting skin repair, followed by several living cellular systems consisting of inorganic particles, polymers, and living cells proposed in current studies. Within each section, some of the most recent advances in application of inorganic biomaterials for treatment of cutaneous wounds are proposed. Then we discuss the main fabrication methods of inorganic particles‐containing biomaterials for skin regeneration. Finally, the conclusion and outlook of the application of the inorganic particle‐based biomaterials in skin tissue engineering are provided.

## 2 INORGANIC BIOMATERIALS USED FOR WOUND HEALING DIRECTLY

With high bioactivities and biocompatibility, some bioactive inorganic biomaterials can be applied alone to treatment of cutaneous wounds in a form of fine powders, microfibers, or nanoparticle suspension.

### 2.1 Bioglass (BG) particles/fibers

There are three main types of BG: silicate‐based glasses, phosphate‐based glasses, and borate‐based glasses.^[^ ^16^ ^]^ BG is originally developed for the repair of hard tissues such as bones and teeth. In the past few decades, BG has been determined to have capacity of stimulating the secretion of growth factors (vascular endothelial growth factor (VEGF) and basic fibroblast growth factor (bFGF)) in cells and promoting soft tissue healing with antibacterial effect in previous studies.^[^ ^17^ ^]^ These biological impacts are induced by the bioactive ions released from BG.^[^ ^16^, ^18^ ^]^ Furthermore, trace elements‐doped BGs are being increasingly concerned to have multiple functions in wound healing. For example, Zhao et al. designed an inorganic wound dressing based on Cu‐doped borate BG microfibers.^[^ ^19^ ^]^ The yielded 3Cu‐BG microfibers with a diameter in the range of 0.4–1.2 μm looked like cotton wool. The results of in vivo study indicated the positive effects of 3Cu‐BG on vascularization, collagen deposition, and wound healing of full‐thickness skin defects. In another study, silver‐containing nanoporous BG (n‐BGS) was fabricated and applied in treatment of skin injury by Hu and coworkers.^[^ ^20^ ^]^ The low content of Ag contributed to antibacterial effect of n‐BGS without cytotoxicity. Besides, the high surface area (467 m^2^/g) of n‐BGS powder results in its practical performance as hemostatic wound dressings with great water absorption efficacy.

### 2.2 Silica‐based particles

Various silica‐based biomaterials attract great interest in skin tissue repair due to the bioactive reaction between particles and tissue.^[^ ^21^ ^]^ In particular, mesoporous silica nanoparticles (MSN) have been widely used in biomedical engineering due to the high specific surface area, organized porosity, and large pore volume.^[^ ^22^ ^]^ For wound healing, the uniform mesoporous silica nanoparticles decorated with ceria nanocrystals (MSN‐Ceria) were synthesized and used as a ROS‐scavenging tissue adhesive by Wu and Li.^[^ ^23^ ^]^ Encouragingly, the full‐thickness skin wound treated with MSN‐Ceria dispersion performed reduced inflammatory response, limited scar formation, and rapid wound closure, resulting from the reduced ROS level and the nanobridging effect between nanoparticles and wound bed. In another study, Dai et al. prepared silver exchanged calcium‐doped mesoporous silica spheres (AgCaMSS) with satisfactory biodegradation rate, hemostatic efficacy, and antibacterial activity, attributing to the addition of Ag and Ca elements in silica spheres.^[^ ^24^ ^]^ As a result, the mesoporous silica‐based materials were proved to promote blood clotting in injuries of animal model.

### 2.3 Metal/metal oxide nanoparticles

Calcium serves as a key regulator of cellular functions involving in dynamic equilibrium of dermal and epidermal reconstruction.^[^ ^25^ ^]^ Therefore, calcium‐based nanoparticles (CNPs) which directly deliver Ca element to wound site have been confirmed to have a therapeutic benefit to skin wound healing by Kawai et al.^[^ ^26^ ^]^ The pH‐sensitive CNP nanoparticles dissolved and released Ca ions in response to the acidic microenvironment of the wound site without changing the total serum calcium levels. Besides, Xu treated infected skin wounds with dispersion of Au nanoparticles‐loaded hydroxyapatite nanorods and achieved good results.^[^ ^27^ ^]^ Furthermore, a mixed solution of palladium (Pd) and platinum (Pt) nanoparticles was reported to be applied to treat skin pathologies in aged mice by Shibuya.^[^ ^28^ ^]^ In addition to the above, Cu nanoparticles concentrations have also attracted interest to repair cutaneous wounds. In the study of Alizadeh, the optimal diameter and concentration of Cu nanoparticles were determined for direct wound treatment in rat model.^[^ ^29^ ^]^

There are certain kinds of metal oxide nanoparticles such as CeO_2_ and Tb_4_O_7_ can be applied to skin repair independently as well. For example, Chigurupati and coworkers provided a study that cerium oxide nanoparticles (3–5 nm) were uniformly dispersed in deionized water and then injected into the full‐thickness skin defect.^[^ ^30^ ^]^ The migration and proliferation of fibroblasts, keratinocytes, and vascular endothelial cells were promoted by Nanoceria, coupled with reduced oxidative stress, leading to rapid skin regeneration. More recently, Tb_4_O_7_ NPs were proved to serve as a potential candidate of antibacterial agents for infected wound healing.^[^ ^31^ ^]^ Of course, no obvious organ damage and cytotoxicity were observed both in vivo and in vitro, which confirmed the biosafety of Tb_4_O_7_ NPs treatment.

Notably, the types of inorganic biomaterials that can be directly applied for skin repair are limited, mainly due to the significant mismatch of mechanical properties between the “hard” inorganic particles and the “soft” skin tissue.^[^ ^15^ ^]^ It cannot be ignored that the degradation rate of inorganic biomaterials in wound bed should be compatible with skin regeneration to avoid tissue calcification occurring. Therefore, only in certain cases can specific inorganic biomaterials be used alone for wound healing. In addition, the long‐term impact of inorganic biomaterials on tissues outside of the wound bed needs to be assessed critically.

## 3 INORGANIC PARTICLE‐BASED COMPOSITE BIOMATERIALS FOR SKIN REGENERATION

In the past few years, the incorporation of inorganic fillers into polymer matrix has been developed as an innovative approach to endow the tissue‐engineered scaffolds with multiple functionalities in order to meet new clinical challenges.^[^ ^32^ ^]^ Typically, two groups of organic materials, natural polymers, and synthetic polymers, are considered to be used for fabricating inorganic/organic composite biomaterials. The polymer materials are required basically to be biocompatible and biodegradable with ample mechanical strength.^[^ ^33^ ^]^ It is emphasized that the degradation of the polymer should match the growth rate of skin tissue. On one hand, natural polymers such as collagen, hyaluronic acid, chitosan, alginate‐based substrates, gelatin, fibrin, and microbial cellulose have become crucial candidates for the matrix material of the composite scaffolds.^[^ ^11^, ^12^, ^14^, ^34^ ^]^ In addition, synthetic polymers including poly(lactic acid) (PLA), poly(glycolic acid) (PGA), poly(L‐lactic acid) (PLLA), poly(vinyl alcohol) (PVA), poly(lactic acid‐co‐glycolic acid) (PLGA), poly(ethylene glycol) (PEG) and poly(ε‐cprolactone) (PCL), are also widely applied to skin reconstruction, owing to the controllable physical and chemical properties.^[^ ^12^, ^35^ ^]^ The polymers can not only cover the wound beds to protect and adhere tissues, but also serve as a vehicle to transfer bioactive molecules to cells.

With the aim of promoting skin repair, wide range of inorganic biomaterials such as (1) bioceramics materials (e.g., bioactive glass, hydroxyapatite, clays, silica, silicates), (2) metal/metal oxides (e.g., gold, silver, platinum, zinc oxide, titanium oxide), and (3) carbon‐based materials (e.g., graphene, carbon nanotubes (CNTs), carbon nanoparticles (CNPs), carbon dots (CDs), nano diamonds) are incorporated in the polymeric matrix to fabricate composite scaffolds.^[^ ^15^, ^36^ ^]^ By this way, the bio‐composite scaffolds can hold modified thermal, mechanical, chemical, or biological characteristics.^[^ ^14^ ^]^ As a result, developing the advanced multi‐functional inorganic particle‐based composite biomaterials can open up new possibilities in various applications for wound healing and skin regeneration.

### 3.1 Bioceramics‐based composite materials

#### 3.1.1 Bioactive glass‐containing composites

**FIGURE 2:**
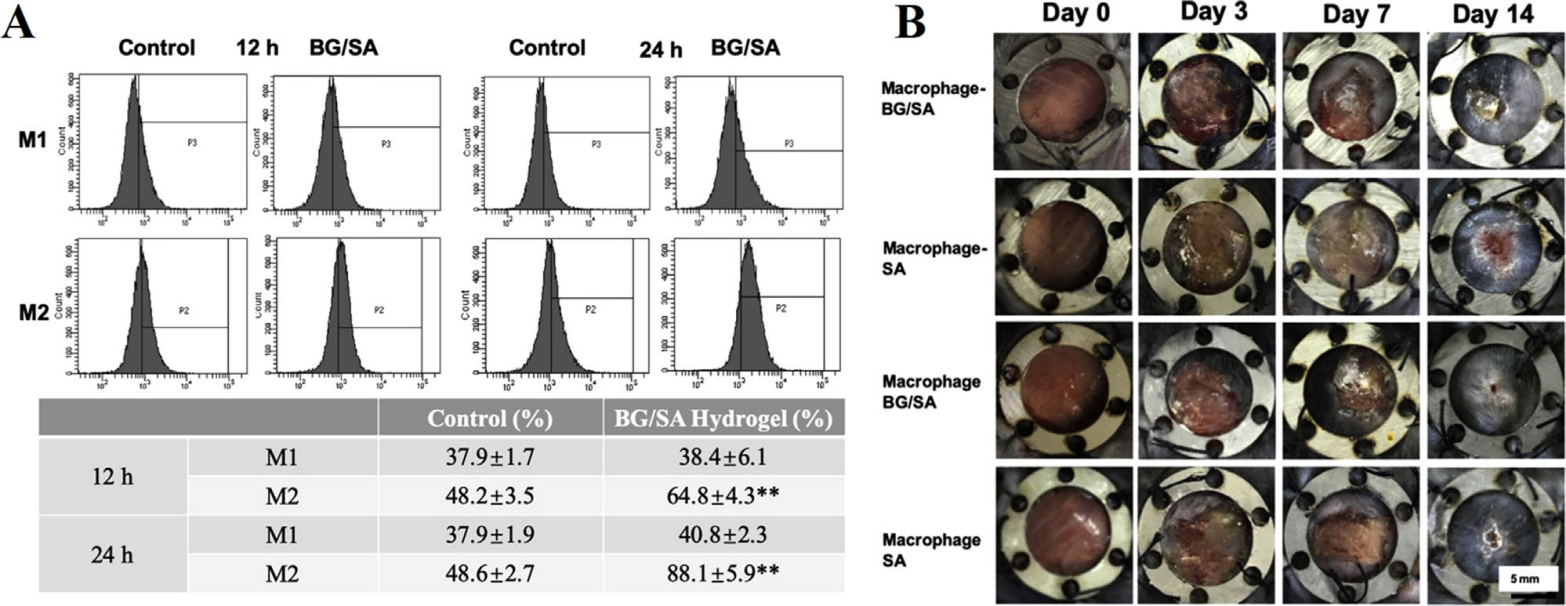
The bioactive glass (BG)/sodium alginate (SA) composite hydrogels modulated the inflammatory responses for efficient skin regeneration. (A) FACS results of RAW cells cultured with normal medium (control) and BG‐containing composite hydrogel after 12 and 24 h. RAW cells were stained with iNOS (M1 marker) and ARG (M2 marker). The table showed the ratios of M1 and M2 phenotypes of the RAW cells. (B) Gross photographs of the skin wounds treated with BG/SA composite hydrogel and SA solution (without BG) in the normal mouse (Macrophage) and macrophage‐depleted mouse (Macrophage‐) at different points of time. Reproduced with permission.^[^ ^39^ ^]^ Copyright 2020, Elsevier

As typical inorganic biomaterials in the field of hard tissue engineering, BGs are gradually extended to be incorporated into polymeric matrix to form flexible composite wound dressings to meet the requirements of soft skin regeneration.^[^ ^37^ ^]^ Taking advantage of the high bioactivities of BG to cells, the BG‐containing composites are confirmed to possess hemostatic, antibacterial, and anti‐inflammatory properties, as well as the capability of enhancing keratinocyte function and angiogenesis.^[^ ^38^ ^]^ Recently, Zhu et al. highlighted the positive effects attributed to the biomaterial‐immune system interaction on wound healing (Figure 2).^[^ ^39^ ^]^ According to their study, the bioactive glass (BG)/sodium alginate (SA) composite hydrogel provided benefit to polarization of the macrophages towards M2 phenotype. As a result, the expression of anti‐inflammatory genes was upregulated, followed by the activation of angiogenesis and ECM deposition. Interestingly, BG/SA hydrogel performed weaker therapeutic effects on wound healing when it activated repairing cells directly in the absence of macrophages than that in the presence of macrophages, indicating the immunomodulatory functions of BG biomaterials. In another study, BG was incorporated in oxidized sodium alginate (OSA) to endow the hydrogel with high bioactivities and tissue adhesiveness for wound healing.^[^ ^40^ ^]^ In addition to hydrogels, BGs can be incorporated into polymer fibers, Vaseline ointments to cope with skin tissue damage.^[^ ^41^ ^]^

It is worth noting that the particle size and porosity are important factors that can significantly affect the properties of BG. With the development of sol‐gel methods, mesoporous bioactive glass and bioactive glass nanoparticles (BGNs) have become attractive materials in the field of tissue engineering due to their uniform morphology, large specific surface area, and controllable pore structure.^[^ ^18^, ^42^ ^]^ One of the outstanding advantages of BG is that the composition is easy to control, so that a variety of elements (Sr, Mg, Ca, Ag, Co, Ga, Zn, etc.) can be easily incorporated into BG network.^[^ ^43^ ^]^ For treatment of skin wound in vivo, it has been reported that Zn,^[^ ^44^ ^]^ Cu,^[^ ^45^ ^]^ Ag,^[^ ^46^ ^]^ Co,^[^ ^41b^ ^]^ and Ce^[^ ^47^ ^]^ elements have been introduced into BG to further improve bioactivities of the BG‐containing wound dressings. Currently, Niu and coworkers developed a mesoporous Eu‐Gd‐Si‐Ca bioactive glass nanoplatform (EGBBGN) integrating with multi‐functions of tumor imaging, inhibiting tumor recurrence and promoting skin regeneration.^[^ ^48^ ^]^ The diameter of branched spherical EGBBGNs was in the range of 150–250 nm. After decorated by FAAL molecule, the EGBBGNs were mixed into the Pluronic F127 hydrogel to form wound dressing for full‐thickness skin defect repair. The composite hydrogel significantly promote the collagen deposition and vascularization of regenerated skin tissue by releasing bioactive Si and Eu ions from the modified BG particles. The introduction of different elements endows BG with enhanced biological properties, such as antibacterial and angiogenic activities. At present, this method has attracted widespread attention and has been widely used in the field of tissue engineering. Therefore, BG still plays an important role in accelerating skin tissue repair.

#### 3.1.2 Silicates‐containing composites

**FIGURE 3:**
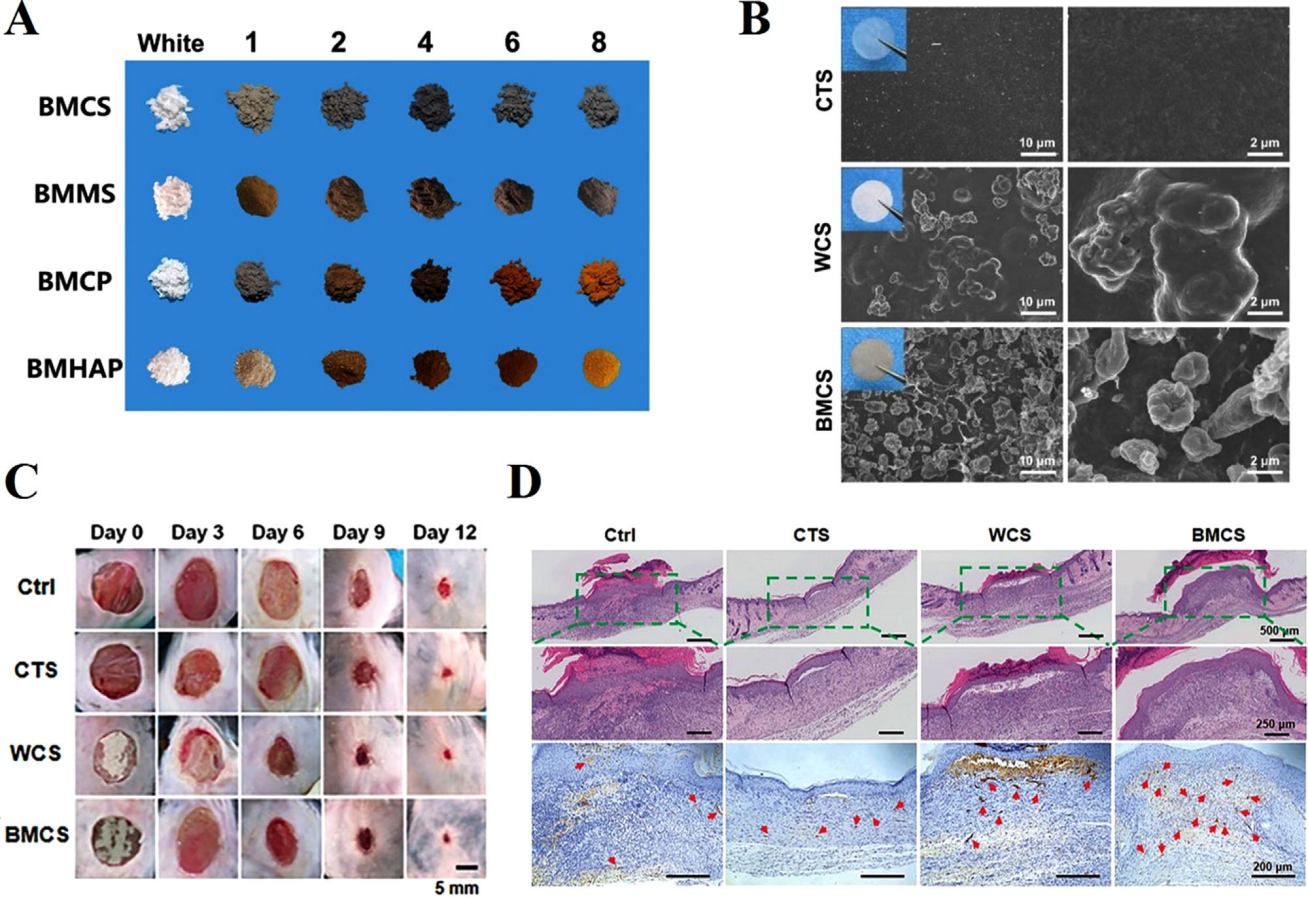
The black CaSiO_3_ bioceramics (BMCS)‐chitosan (CTS) membranes for skin wound healing. (A) Photographs of bioceramics of black CaSiO_3_ (BMCS) powders obtained from the magnesium thermal reaction with increasing contents of Mg powders (the first line) as compared to the original white BMCS. (B) SEM images of pure CTS, WCS‐ (white CaSiO_3_), and BMCS (black CaSiO_3_)‐CTS membranes at different magnifications. (C) Representative gross photos of chronic wounds with different treatments at days 0, 3, 6, 9, and 12. (D) The images of staining of H&E and α‐SMA of the wound sections from samples in different groups on day 12. The blood vessels in skin showed α‐SMA positive in brown (red arrows) and cell nuclei showed in blue. Reproduced with permission.^[^ ^51^ ^]^ Copyright 2020, Wiley‐VCH

As an essential constant element in human body, Silicon (Si) shows a positive effect on blood vessels formation and collagen deposition during wound healing process.^[^ ^49^ ^]^ In recent years, various silicates have been applied to enhance biofunctions of composite wound dressings through releasing bioactive Si and metal ions. Previously, the effects of calcium silicate (CS) on expression of vascular‐related genes in co‐cultured fibroblasts‐endothelial cells system have been demonstrated in literature.^[^ ^50^ ^]^ In a recent study of our group, novel black CaSiO_3_ bioceramics (BMCS) was synthesized via a magnesium thermal reaction method, and then the BMCS powders were mixed into chitosan (CTS) matrix to form composite membranes for chronic wound healing.^[^ ^51^ ^]^ Related to the releasing of Si and Mg bioactive ions, the outstanding angiogenesis activity of the BMCS‐CTS composite membrane was discovered during the wound‐healing process in diabetic mice. As a result, the black‐bioceramic‐incorporating composite membranes possessed satisfactory capacity in promoting skin regeneration (Figure 3). In another work, Zhang et al. reported a unique sandwich‐structured organic/inorganic wound dressing, which was fabricated via hot compression molding using zinc silicate bioceramics (hardystonite, Ca_2_ZnSi_2_O_7_, ZnCS) and PLA.^[^ ^52^ ^]^ As known, severe cutaneous wounds are always accompanied by damage of hair bulbs, so that few remnants of follicle stem cells remain in the dermis.^[^ ^53^ ^]^ As a result, the non‐renewable hair follicles cannot maintain hair growth in the repairing skin tissue. For the treatment of deep burn skin injury, the ZnCS‐containing composite membrane could absorb wound exudates rapidly and release Zn^2+^ and SiO_3_^2–^ bioactive ions. Thus, the positive effects of the composite membrane on hair follicle regeneration and skin repair were confirmed in the third‐degree scald mouse models. Similarly, a hardystonite‐containing composite hydrogel with antibacterial property was applied to promote angiogenesis and wound healing by Li et al.^[^ ^54^ ^]^ Apart from the types of materials listed above, there are some other silicate‐based bioceramic particles such as Nagelschmidtite (NAGEL, Ca_7_P_2_Si_2_O_16_) can serve as bioactive fillers in composite wound dressings.^[^ ^55^ ^]^

In addition, silicate/silica nanostructures used for wound healing have also been investigated.^[^ ^56^ ^]^ Silicate nanoparticles can not only delivery proteins and drugs to the wound bed, but also release bioactive ions in a controlled manner to stimulate skin cells. For instance, copper silicate hollow microspheres (CSO HMSs) were fabricated and incorporated into polymer electrospun scaffold by Yu et al.^[^ ^36h^ ^]^ The composite scaffolds had the dual functions of melanoma therapy and repair skin defects. Besides, a CaCuSi_4_O_10_ nanoparticles‐containing scaffold prepared using a similar method was also proven to have great potential for both tissue regeneration and tumor therapy.^[^ ^57^ ^]^ In another study on the composite hydrogel with incorporation of manganese silicate hollow nanospheres (MS HNSs), Ma et al. confirmed that the bioactive Mn and Si ions promoted the recovery rate of diabetic wound model.^[^ ^58^ ^]^ The release of the bioactive ions of silicates is the focus of their wide application in the field of skin tissue engineering. The combination of silicates and polymers can help to achieve the purpose of slowing down the release rate of ions controllably, so as to continuously stimulate the cell activities, and then the skin regeneration can be promoted.

#### 3.1.3 Nanoclays‐containing composites

Clay, especially the smectite family, has been proved to possess a wide range of hemostatic properties for wound healing. The clay materials swell upon hydration after absorbing water, resulting in thickening and clotted blood.^[^ ^59^ ^]^ The adequate hemostatic response after skin injury is recognized to be a prerequisite for biological activities in wound healing, such as immune response, fibrin network formation, and wound edges contraction.^[^ ^60^ ^]^ For example, the development of a halloysite (Al_2_Si_2_O_5_(OH)_4_·2H_2_O)‐containing composite wound dressing has been reported by researchers.^[^ ^61^ ^]^ In a recent work, Li and coworkers designed a halloysite nanotube (HNT)‐incorporated chitosan (CS)/oxidized dextran (ODEX) hydrogel.^[^ ^62^ ^]^ After being injected in wound beds, the composite CS/ODEX/HNT hydrogel could clot within 1 s, leading to rapid hemostasis and reduced blood loss. In the wound‐healing study, the results confirmed that the HNT‐based composite hydrogel contributed to accelerating infected wound closure significantly. Besides, montmorillonite (MMT, hydrous alumina‐silicate clay)‐incorporated composite hydrogels have been reported to be used as cost‐effective wound dressings in previous literature.^[^ ^63^ ^]^ Similarly, montmorillonite modified with Cu, Ca, and Na was blended in bacterial cellulose (BC) to form Cu‐MMT, Na‐MMT, and Ca‐MMT nanocomposites by Sajjad et al.^[^ ^64^ ^]^ The introduction of these elements added activities of antimicrobial and angiogenesis to the modified MMTs‐BC skin substitutes, leading to enhanced tissue regeneration in burn wounds.

Additionally, clays can be used as functional fillers in the polymer network to form a hemostatic system with reinforced mechanical properties.^[^ ^65^ ^]^ In this regard, Han et al. designed a polydopamine‐clay‐polyacrylamide (PDA‐clay‐PAM) hydrogel inspired by mussel adhesion.^[^ ^66^ ^]^ The close interaction between clay nano‐reinforcements and polymers in the hydrogel networks contributed to superior adhesiveness, stretchability, and toughness of the composite wound dressing. In another recent work, Zandi and coworkers designed bilayered scaffolds composed of the upper layer of Laponite (LA) nanoplatelets‐containing hydrogel and the lower layer of gelatin nanofibrous matrix.^[^ ^67^ ^]^ Due to the tunable mechanical, hemostatic, and adhesive properties of LA incorporated hydrogel, the multifunctional biomimetic scaffold had a great performance on repairing excisional wounds in vivo. Similarly, Gaharwar et al. highlighted the significant hemostatic functions of composite hydrogels composed of silicate nanoplatelets and gelatin hydrogel.^[^ ^68^ ^]^ With an ideal shear‐thining property, the nanocomposite hydrogel could be injected into wound sites and coagulate rapidly. In the in vivo study, the applicable hemostatic performance of the silicate‐gelatin composite system was revealed, due to rapid blood coagulation. The research on the application of Laponite nanocomposite hydrogel for skin wound healing was reported by Lokhande et al.^[^ ^69^ ^]^ Moreover, Laponite was also been used as carriers to deliver drugs and growth factors to wound site by the research team of Page.^[^ ^70^ ^]^

#### 3.1.4 Hydroxyapatite‐containing composites

**FIGURE 4:**
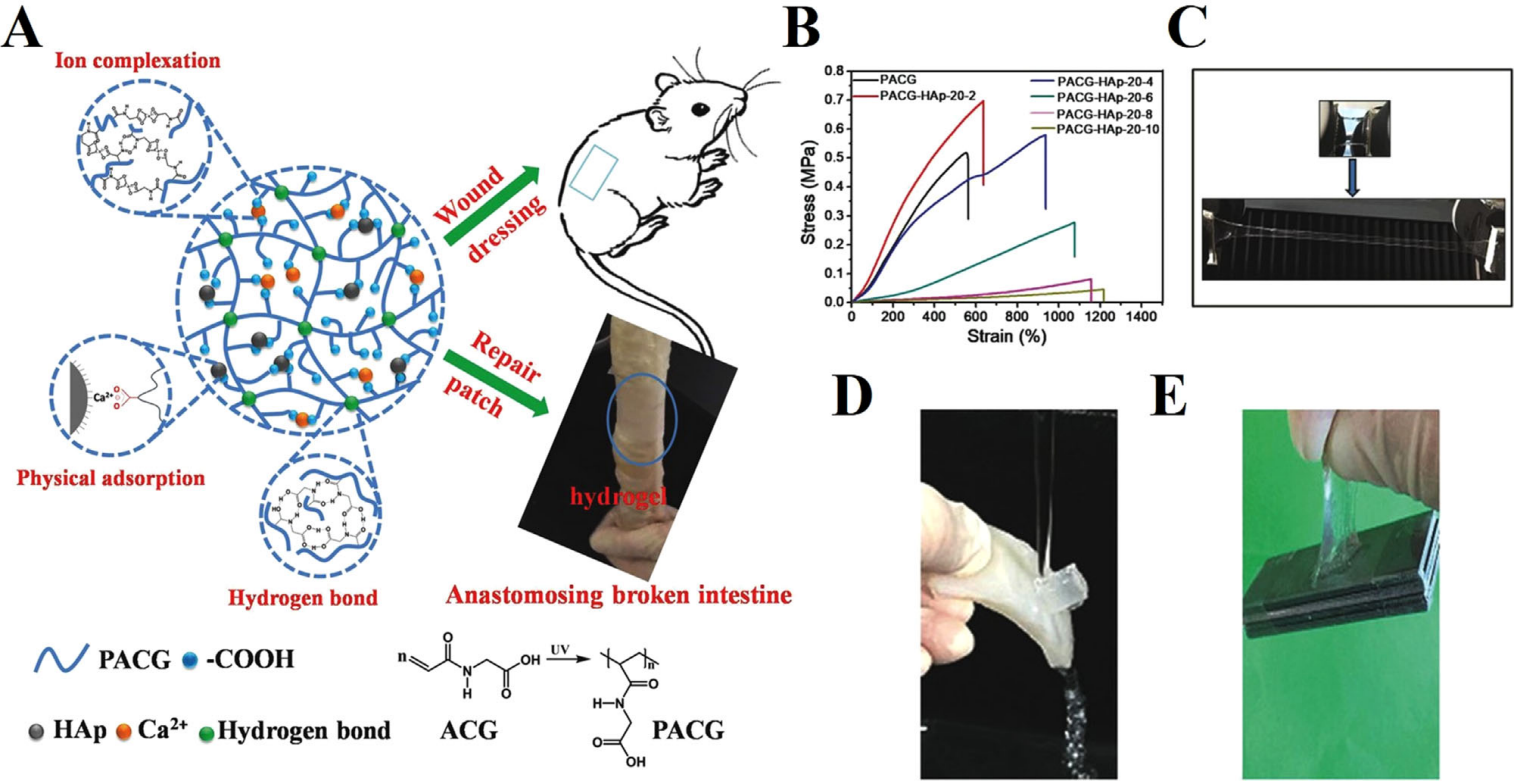
The hydroxyapatite‐incorporated organic–inorganic hybrid (OIH) hydrogel with high strength and adhesion for wound healing. (A) Schematic illustration of the formation of the HAp‐based OIH hydrogel and its application for soft tissue adhesive. (B) The curves of stress–strain of the composite PACG‐HAp‐20‐X hydrogels. (C) The photo showed the ability of the composite PACG‐HAp‐20‐10 hydrogel to withstand stretching under the strong external force. (D) Photographs illustrated the great adhesion of the HAp‐incorporated composite hydrogel to surface of porcine skin under water flushing. (E) The photo exhibited that the PACG‐HAp‐20‐6 hydrogel adhered to the iron sheets with a weight of 100 g rapidly (100 time of the gel weight). Reproduced with permission.^[^ ^71^ ^]^ Copyright 2018, Wiley‐VCH

As a compound in calcium phosphate family, hydroxyapatite (HAP) has become widespread in hard tissue engineering. Nevertheless, the integration of nano‐hydroxyapatite and polymers expands its application field to soft tissue repair. Cui et al. fabricated an organic–inorganic hybrid (OIH) hydrogel by polymerization of N‐acryloyl 2‐glycine (ACG) with addition of hydroxyapatite.^[^ ^71^ ^]^ Remarkably, due to PACG chain‐HAp physical interactions, the PACG‐Hap hydrogels hold prominent adhesive, self‐healing, and mechanical properties (adhesion strength of 105 kPa, tensile strength of 0.72 MPa, compressive strength of 5 MPa, break strain of 1200%) (Figure 4). In other works, nanohydroxyapatite was incorporated into polymers (chitosan, collagen, gelatin, etc.) to form antibacterial dressings to reduce inflammatory responses and promote epithelialization in chronic wound beds.^[^ ^72^ ^]^

HAP can also be modified into metal elements such as Zn, Ag, Co, Cu, Bi, and Sr to obtain enhanced antibacterial activity.^[^ ^73^ ^]^ In this way, Pyun et al. fabricated AgHAP‐incorporated wound dressings for the treatment of infected wounds.^[^ ^74^ ^]^ A cobalt‐substituted hydroxyapatite (CoHAP)‐polyvinyl alcohol (PVA) nanocomposite hydrogel was proposed in the study of Lin.^[^ ^75^ ^]^ As a result, the composite wound dressings containing modified HAP can display desired functions for rapid wound healing.

### 3.2 Metal/metal oxide‐based composite materials

#### 3.2.1 Metal nanoparticles‐containing composites

Bacterial infection is considered to be one of the major challenges for clinic treatment of cutaneous wounds.^[^ ^76^ ^]^ Introducing antibacterial agents to polymeric networks can be of great benefit to combating bacterial infections in wound sites.^[^ ^77^ ^]^ As known, metal nanoparticles (AuNPs, AgNPs, CuNPs, SeNPs, etc.) exhibit attractive antibacterial properties for wound healing according to previous literature.^[^ ^36^, ^78^ ^]^ Au nanoparticle (AuNP) is widely used in various biomedical applications due to its biocompatibility, anti‐microbial activity, and angiogenic function.^[^ ^79^ ^]^ Notably, the attachment of chemical compounds and biomolecules to the surface of AuNPs has become the common adoption of skin disease treatment.^[^ ^80^ ^]^ Therefore, the great potential of AuNPs as therapeutic anti‐oxidative agents in treatment of diabetic wounds has been discovered.^[^ ^81^ ^]^ For instance, Li et al. synthesized and decorated 4,6‐diamino‐2‐pyrimidinethiol‐modified gold nanoparticles (Au‐DAPT NPs) on bacterial cellulose (BC) membrane to obtain BC‐Au‐DAPT nanocomposites for bacteria‐infected wound healing.^[^ ^82^ ^]^ The BC‐Au‐DAPT composite system showed robust multidrug‐resistant (MDR) bacteria inhibition effects and high biocompatibility. In another work, a wound dressing was prepared by mixing AuNPs with gentamicin sulfate, konjac glucomannan, and gelatin.^[^ ^83^ ^]^ In addition, researchers take advantage of AuNPs conjugated with platelet derived growth factors or VEGFs to delivery bioactive molecules to wound site.^[^ ^84^ ^]^

Ag nanoparticle (AgNP) is one of the most viable nanoparticles participating in wound management. AgNPs were developed to replace traditional silver compounds to reduce tissue toxicity due to the high bioactivities in low dose.^[^ ^36c^ ^]^ Choudhury et al. highlighted the application of AgNPs in diabetic wound therapy and discussed the mechanism of AgNP‐cell interaction.^[^ ^85^ ^]^ Recently, El‐Aassar and coworkers suggested a nanofibrous dressing consisting of AgNPs, polygalacturonic acid (PGA), and hyaluronic acid (HA).^[^ ^86^ ^]^ The results of the study confirmed the improved antibacterial activity and hydrophilicity provided by the AgNP‐based composite dressing for quick wound repair. In another study, a composite hydrogel composed of ultrasmall AgNPs was fabricated by Haidari et al.^[^ ^87^ ^]^ The uncontrolled release of Ag ions from the thermo‐responsive composite hydrogel led to rapid wound recovery with low tissue toxicity. Anisha et al. prepared an antibacterial sponge containing nano silver for treatment of infected diabetic foot ulcers.^[^ ^88^ ^]^ However, many studies showed that the production of ROS in wound bed could be increased result from AgNPs‐induced DNA damage and inflammation response.^[^ ^89^ ^]^ Hence, it cannot be ignored that the cytocompatibility and tissue toxicity of AgNPs‐based wound dressings for wound treatment should be strictly evaluated.

Composite wound dressings containing CuNPs or SeNPs are chosen for wound repair as well. The photothermal performance of CuNPs endows the wound dressings with predominant antibacterial efficacy. In the meanwhile, the released Cu ions can stimulate fibroblast proliferation and angiogenesis through regulating factors involved in skin repair process.^[^ ^90^ ^]^ Gopal et al. combined chitosan and CuNPs to obtain a nanocomposite for the treatment of excision wounds.^[^ ^91^ ^]^ The results of in vivo study indicated high wound contraction, angiogenesis, and collagen deposition that attributed to CuNPs. Besides, CuNP‐embedded hydrogels possess great potential in accelerating skin regeneration in the bacteria‐infected wounds as well.^[^ ^90^ ^]^ In another recent study, Mao et al. reported a multifunctional nanocomposite biomaterial based on selenium nanoparticles (SeNPs) and bacterial cellulose (BC)‐gelatin (Gel) hydrogel.^[^ ^92^ ^]^ The embedded SeNPs with a sustainable release profile endowed the composite hydrogel with outstanding antioxidant, anti‐inflammatory, and antibacterial capabilities. As a result, the BC/Gel/SeNPs hydrogels exhibited great performance in skin wound healing of full‐thickness defects.

#### 3.2.2 Metal oxide nanoparticles‐containing composites

**FIGURE 5:**
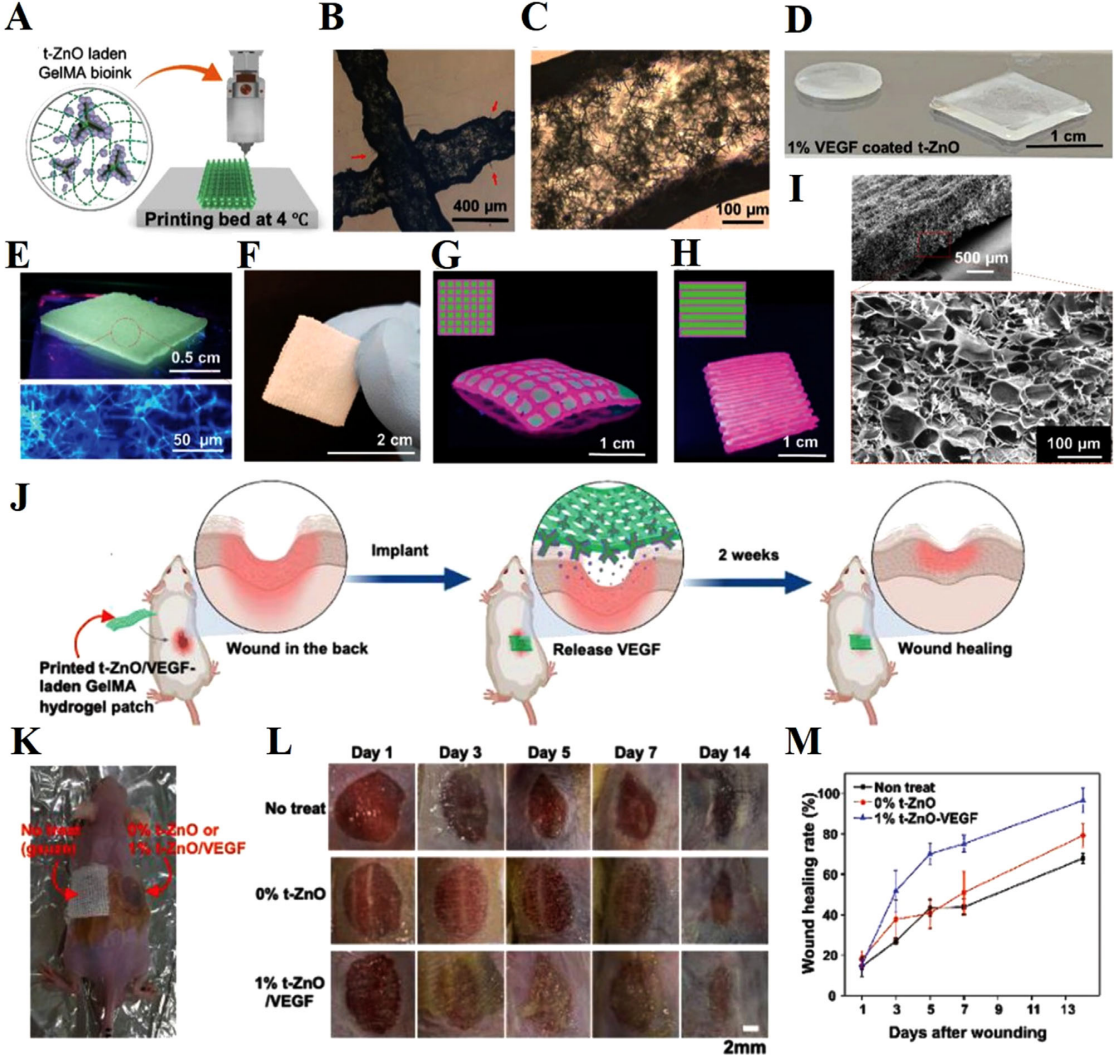
3D printing of t‐ZnO‐incorporated composite hydrogel constructs. (A) Schematic diagram of the printing of t‐ZnO‐incorporated composite ink at 4°C and crosslinked by UV light exposure. (B) Phase contrast images of the 3D‐printed 10% t‐ZnO‐incorporated composite hydrogels and (C) well‐dispersed t‐ZnO in the filaments. (D) Different shapes of the t‐ZnO‐laden composite hydrogel. (E) Photograph of the 3D printed construct under UV and the confocal micrograph of the 2% t‐ZnO‐incorporated construct. (F) Photograph of the printed composite construct with excellent mechanical stability. (G,H) Photographs of the 3D printed constructs with different arrangements using two colored t‐ZnO‐incorporated composite inks with multi‐nozzle printer for synergetic effect of two inks. (I) SEM image of the microporous morphology of the printed construct and incorporated t‐ZnO microparticles. (J) Schematic illustration for in vivo wound healing process. (K) Representative photos of wound healing operation. (L) Photos of the murine skin wounds treated with no treatment, 0% t‐ZnO, and 1% t‐ZnO‐VEGF. (M) The healing rate of murine wounds measured at different points of time. The treatment of 1% t‐ZnO‐VEGF significantly accelerated wound healing compared with the other groups. Reproduced with permission.^[^ ^95^ ^]^ Copyright 2021, Wiley‐VCH

ZnO nanoparticles play a crucial role in the application of wound healing due to their antimicrobial activity and anti‐inflammatory.^[^ ^93^ ^]^ Various types of ZnO nanoparticles‐incorporated composite wound dressings have been shown in previous studies. For instance, Ahmed et al. developed a nanofibrous membrane composed of chitosan, polyvinyl alcohol, and ZnO particles, and another study of composite beads of similar composition was reported by Gutha.^[^ ^94^ ^]^ Recently, Siebert et al. developed a 3D printed t‐ZnO‐laden hydrogel patch encapsulating VEGF.^[^ ^95^ ^]^ In this study, ZnO was modified into t‐ZnO particles and decorated with VEGF. The elastic modulus and degradation rate of the printed patch can be adjusted by changing the t‐ZnO content in composite hydrogel. Through in vivo study, the t‐ZnO‐incorporated composite hydrogel was confirmed to have controllable molecular release behavior, low cytotoxicity, antibacterial function, and high angiogenic activity to support fast wound healing (Figure 5). Moreover, ZnO can be selected as a piezoelectric material with great biocompatibility in dermal patch for electrical field therapy. Bhang and coworkers reported a ZnO nanorod‐based piezoelectric patch which promoted cell proliferation, angiogenesis, and skin tissue reconstruction through supplying exogenous electrical field in wound beds.^[^ ^96^ ^]^

Likewise, titanium oxide (TiO_2_) nanoparticles‐containing composites are also promising candidates with outstanding antibacterial activities for treatment of skin defects. For example, Razali et al. prepared a TiO_2_ nanotubes‐incorporated gellan gum bio‐nanocomposite film for wound healing.^[^ ^97^ ^]^ Niranjan et al. combined TiO_2_ nanoparticles and curcumin to obtain TiO_2_‐Cur nanocomposite hydrogel patch with antimicrobial and antioxidant property.^[^ ^98^ ^]^ Similarly, the synergistic effect of curcumin and TiO_2_ on treatment of infected wound was detected by the team of Marulasiddeshwara.^[^ ^99^ ^]^

Cerium oxide nanoparticles possess antioxidant capacity to scavenge radicals.^[^ ^100^ ^]^ It has been confirmed that cerium oxide nanoparticles can stimulate the proliferation of keratinocytes and fibroblasts, as well as the bioactivities of vascular endothelial cells.^[^ ^30^ ^]^ Therefore, the therapeutic potential for wound treatment with antioxidant cerium oxide nanoparticles worthy of further exploration. Recently, Augustine et al. proposed a cerium oxide (CeO_2_) nanoparticles‐containing electrospun membrane.^[^ ^101^ ^]^ The synthesized nCeO(2)‐PHBV composite membranes were confirmed to enhance dermal vascularization and accelerate wound healing in diabetic rat models. In another study, Cheng and Shi designed a sprayable hydrogel wound dressing loaded with cerium oxide nanoparticles.^[^ ^102^ ^]^ Based on the results of in vivo study, the composite hydrogel dressing with favorable ROS‐scavenging ability played a dominant role in chronic wound treatment and management.

Terbium (Tb), a rare earth element, has emerged as a promising agent for tissue engineering currently. It is reported that terbium hydroxide nanorods (THNs) possess the capacity of inducing ROS production, NO formation, and angiogenesis.^[^ ^103^ ^]^ After treated with THNs‐containing Vaseline cream, murine skin wounds exhibited rapid healing rate, which could be attributed to the outstanding pro‐angiogenic property of Tb nanorods.

Apart from the metal oxide biomaterials cited above, CuO, Fe_3_O_4_, α‐Fe_2_O_3_, γ‐Fe_2_O_3_ nanoparticles were also reported to be incorporated into composites for efficient hemostasis, antibiosis, and skin regeneration.^[^ ^104^ ^]^ For example, the thrombin‐conjugated γ‐Fe_2_O_3_ nanoparticles were confirmed to be conductive to rapid incisional wound healing by Ziv‐Polat et al.^[^ ^105^ ^]^ Iron oxides are available in tissue engineering for loading and delivering biomolecules because of their biocompatibility and diverse morphology such as nanoparticles, nanotubes, nanofibers, and nanoflowers.^[^ ^106^ ^]^

### 3.3 Carbon‐based composite materials

Graphene is a single layered carbon sheet with unique properties, presenting surprised opportunities for tissue engineering.^[^ ^107^ ^]^ With regard to biomedical researches of graphene, widespread concern has been aroused toward graphene/graphene oxide‐incorporated composite dressings. On one hand, the available antimicrobial activity of graphene allows it to be applied to treatment of cutaneous wounds. On the other hand, graphene and graphene derivatives can offer mechanical reinforcement and chemical versatility to the composite wound dressings.^[^ ^108^ ^]^ For example, a strategy for the production of wound dressing with desired mechanical strength was developed by Xue et al.^[^ ^109^ ^]^ The graphene oxide‐chitosan‐calcium silicate (GO‐CTS‐CS) biomaterial with layered structure was prepared by combining the methods of vacuum filtration‐assisted assembly and freeze‐drying. Benefiting from the addition of GO and ordered nanostructure, the hierarchical GO‐CTS‐CS biomaterials possessed significant antibacterial property with enhanced tensile strength up to 10.10 MPa. Thus, the composite biomaterials with light weight and high strength had great potential in treatment of diverse wounds. Fan et al. reported an grapheme‐based composite hydrogel with enhanced swelling ratio and tensile strength.^[^ ^110^ ^]^ In the in vivo study, the great wound‐healing effects of the composite hydrogel wound dressing were confirmed.

CNTs are rolled‐up graphene sheets with a diameter less than 100 nm.^[^ ^107^ ^]^ CNT‐based composites have been studied for wound healing because of their high electrical conductivity, great stability, and enhanced mechanical properties. For example, CNTs were used as addictive to enhance the mechanical strength of macroporous cryogels.^[^ ^111^ ^]^ The CNT‐reinforced composite cryogels showed both outstanding blood‐clotting ability with high blood absorption speed and activation of cellular activity derived from electro‐signals, contributing to rapid skin repair. Notably, CNTs can be introduced into wound dressings as agents for photothermal therapy to eliminate bacterial infection in cutaneous wounds. More recently, He et al. developed a series of CNT‐based composite hydrogels with multiple functions including photothermal property, self‐healing performance, and tissue adhesiveness.^[^ ^112^ ^]^ The hydrogels presented high efficiency and safety to accelerate bacteria‐infected wound closure in vivo.

Carbon dots (CDs) are a type of extremely small carbon nanoparticles. In recent years, CDs to serve as biocompatible fillers in various nanocomposites have been applied in the field of tissue engineering.^[^ ^113^ ^]^ According to previous study, CDs offered benefit to increase of cell motility and reduced inflammatory reaction, leading to rapid re‐epithelialization with few scar formation.^[^ ^114^ ^]^ The remarkable antibacterial activity of CDs‐incorporated composite hydrogels allows them to be applicative for wound repair.^[^ ^115^ ^]^ For example, Li and coworkers developed a CDs‐decorated injectable hydrogel with good biocompatibility, enhanced mechanical performance, and antibiofilm activity.^[^ ^116^ ^]^ As a result, fast wound healing process was induced by the composite hydrogel, coupled with high collagen content. Furthermore, CDs can also be used as pH nanosensors to detect pH value in wound site due to their fast response and high sensitivity. In a recent study, Yang et al. reported a novel orange‐emissive CDs‐coated cotton cloth which exhibited timely response to wound pH value at a range of 5–9.^[^ ^117^ ^]^ Combining with antibacterial activity, the multifunctional CDs‐coated cloth showed great potential in the clinical treatment of diabetic wounds.

Additionally, nano diamonds (NDs), as another member of carbon family, can also be widely used to decorated polymers for tissue engineering. NDs hold high mechanical stability and thermal conductivity, as well as good biocompatibility.^[^ ^118^ ^]^ Houshyar et al. prepared a NDs‐PCL composite nanofibrous scaffold for wound management.^[^ ^119^ ^]^ The hydrophilic groups on the surface of NDs led to efficient cell attachment and applicable moisture management properties of the scaffold. As a result, the PCL‐NDs nanocomposite scaffolds showed improved hydrophilicity and biocompatibility in the study. In another work, Khalid combined NDs and silk to obtain a composite membrane in which the NDs served as nanoscale thermometers for biosensing.^[^ ^120^ ^]^ This design relied on the specific temperature biomarker in chronic wounds. Besides, the addition of NDs enhanced thermal stability of silk without any adverse effects on wound healing process.

### 3.4 Other inorganic biomaterials in composite wound dressing

**FIGURE 6:**
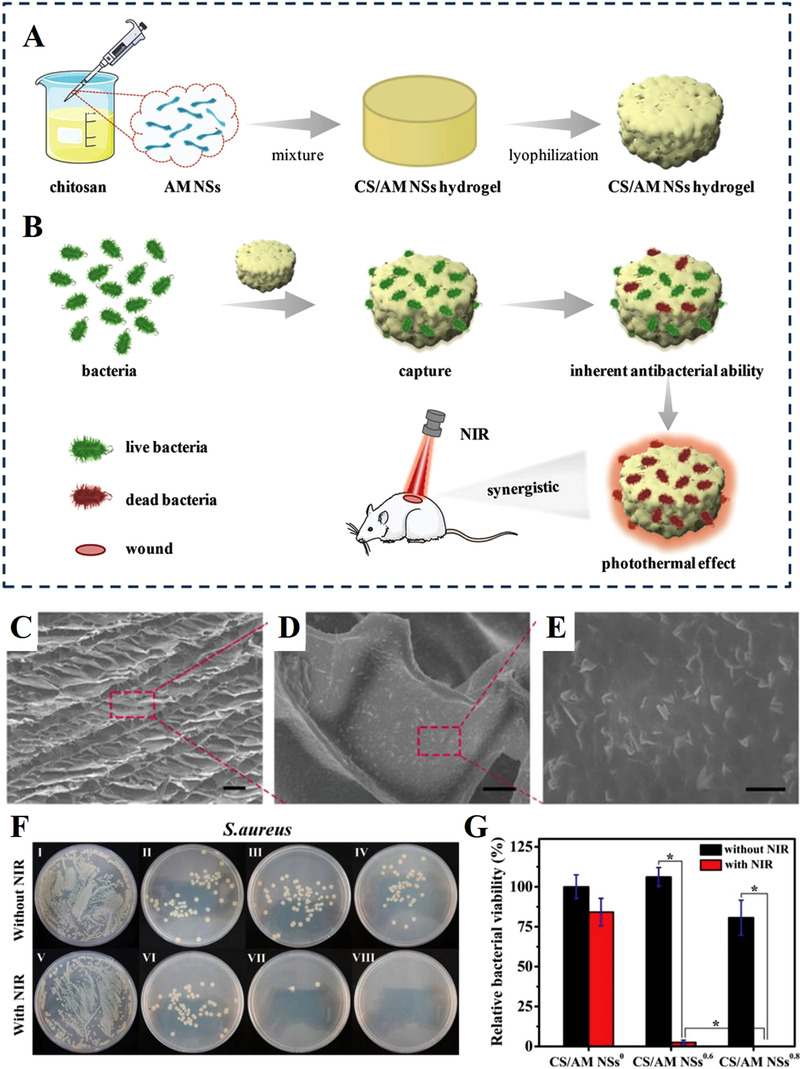
Chitosan‐based composite hydrogel incorporated with antimonene nanosheets (AM NSs). Schematic illustration of (A) the preparation of the composite hydrogel and (B) its application in treating infected wound. (C–E) SEM images of the composite hydrogel containing antimonene nanosheets. (F) Photos of bacterial colonies formed by bacteria after treatments with (I) PBS, (II) NSs^0^ composite hydrogel, (III) NSs^0.6^ composite hydrogel, (IV) NSs^0.8^ composite hydrogel, (V) PBS+NIR, (VI) NSs° composite hydrogel+NIR, (VII) NSs^0.6^ composite hydrogel+NIR, (VIII) NSs^0.8^ composite hydrogel+NIR. (G) The survival rates of the S. aureus bacteria. Reproduced with permission.^[^ ^122^ ^]^ Copyright 2020, Wiley‐VCH

In addition to the above, there are some other types of inorganic biomaterials‐based composite wound dressings developed for skin repair. For example, Liu et al. developed a novel portable electrospinning device to in situ deposit CuS composite nanofibers onto the cutaneous wounds.^[^ ^121^ ^]^ Rapid hemostasis within 6 s was achieved because of the convenient operation and high compactness of composite nanofibers, and then the elapsed time of wound healing was shortened. In another study, Liu et al. took advantage of the capture effect of chitosan (CS) and the photothermal effect of antimonene nanosheets (AM NSs) to eliminate bacteria including *E. coli* and *S. aureus*.^[^ ^122^ ^]^ The CS/AM NSs composite hydrogel was constructed with a blend of antimonene nanosheets and chitosan to serve as a broad‐spectrum antibacterial wound dressing. The results confirmed the satisfactory antibacterial abilities of the AM NSs‐containing composite hydrogel in treatment of infected skin wounds (Figure 6).

Researchers also combined more than two types of inorganic biomaterials with polymer to produce composite wound dressings with multiple biofunctions or improved properties. For example, bimetallic Ag‐Au nanoparticles were developed and coated with carbohydrate by Kumar et al.^[^ ^123^ ^]^ Compared with AgNPs, the composite Au‐Ag nanoparticles showed enhanced stability and antibacterial property during the process of infected wound healing. Besides, Wang et al. reported a novel antibacterial film consisting of PVA, ZnO nanoparticles, CuO nanoparticles, and AuNPs.^[^ ^124^ ^]^ The composite films could not only display photothermal and photodynamic effects to eliminate bacteria but also promote vascularization through releasing Zn^2+^ and Cu^2+^. Zhu and coworkers adopted a method of introduction of 2D Ti_3_C_2_T*_x_* MXene in the Ag NPs‐containing composite hydrogel.^[^ ^125^ ^]^ The Ti_3_C_2_T*_x_* MXene not only reduced the cytotoxicity of the high dosage of Ag NPs but also offered enhanced photothermal therapy (PTT)‐associated antibacterial effects. Therefore, the Ag/Ti_3_C_2_T*_x_* embedded hydrogel exhibited remarkable antibacterial function in the animal study.

## 4 INORGANIC BIOMATERIALS‐CONTAINING CELLULAR ENGINEERED LIVING SYSTEM FOR SKIN REGENERATION

With the rapid development of bio‐manufacturing technology, more complex and sophisticated biomaterial systems are being developed for skin tissue engineering.^[^ ^126^ ^]^ Recent studies have increasingly established cellular living systems incorporating inorganic particles to further stimulate skin regeneration, both chemically and biologically. In this way, the engineered composite system loading with multiple skin cells can serve as a platform to deliver bioactive ions, molecules, and cells to the wound site efficiently with the purpose of constructing a microenvironment conducive to tissue regeneration. However, not only the option of inorganic fillers but also the production methods are limited by the presence of living cells inside the system.^[^ ^127^ ^]^ Therefore, only specific bioactive inorganic materials with high biocompatibility as well as low cytotoxicity can be adopted to fabricate living cellular system for skin regeneration.

### 4.1 BG‐based cellular system

BG nanoparticles have been incorporated in hydrogel to form bioactive composite cell‐laden systems in previous literature. It has been found that the proliferation and adhesion of dermal fibroblasts encapsulated in the BG‐containing hydrogel were promoted by BG nanoparticles.^[^ ^128^ ^]^ Beyond that, Keshaw et al. encapsulated BG particles and fibroblasts into alginate beads to function as bioreactors producing growth factors.^[^ ^129^ ^]^ The BG‐incorporated cellular beads with increased VEGF secretion could benefit to neovascularization which is required for tissue regeneration. Specifically, a cellular skin substitute combining fibroblast and BG was prepared via cell sheet technology by Yu et al.^[^ ^130^ ^]^ On the basis of stimulation of BG to fibroblasts, the bio‐activated cell sheet played a dominant role in enhancing vascularization, collagen deposition during murine wound healing process, leading to rapid skin reconstruction.

### 4.2 Silicate particles‐based cellular system

**FIGURE 7:**
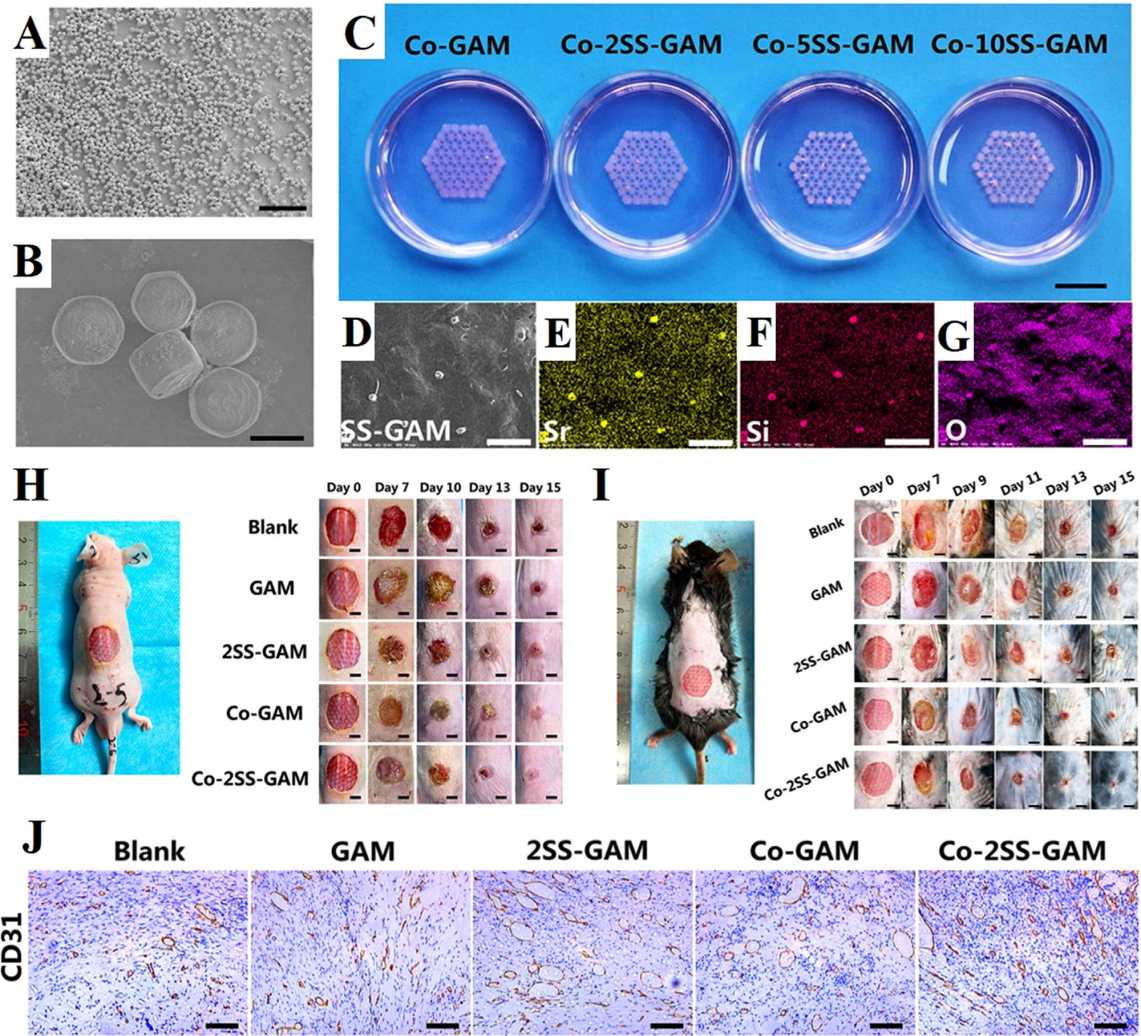
3D bioprinting strontium silicate microparticles‐containing multicellular scaffolds for vascularized skin regeneration. (A,B) SEM images showed the uniform morphology of nearly hexagonal prism of strontium silicate microparticles. (C) Photograph of the 3D bioprinting strontium silicate microparticles‐containing multicellular scaffolds. (D) SEM image of the surface of the scaffold and corresponding EDS elemental mapping of (E) element Sr, (F) element Si, (G) element O. (H) Photos of acute wounds with different treatments at different points of time. (I) Photos of chronic wounds with different treatments at different points of time. (J) Images of immunohistochemical staining with CD31 antibody. The Co‐2SS‐GAM group showed the highest degree of angiogenesis. Reproduced with permission.^[^ ^137^ ^]^ Copyright 2021, Wiley‐VCH

Silicate micro/nanoparticles can be introduced into a cellular system to endow it with enhanced biological activities such as immunoregulation and angiogenesis, which attribute to the stimulation of bioactive ions. Following skin tissue injury, the proper immune response can stimulate cell differentiation, ECM deposition, and secretion of growth factors.^[^ ^131^ ^]^ Nevertheless, the dysregulated inflammation and abnormal immune response in the damaged skin tissue always lead to chronic wounds or scarring.^[^ ^132^ ^]^ To address the issues, our team developed a manganese silicate (MS) nanospheres‐containing bioink for stimulating immune response to promote angiogenesis in tissue engineering.^[^ ^133^ ^]^ Previous research has proved that Mn element can be used as a potential stimulator for immune response both in vitro and in vivo.^[^ ^134^ ^]^ As a result, the MS‐incorporated bioink could promote the expression of several anti‐inflammatory genes and macrophage polarization. Then the MS‐mediated immune microenvironment stimulated endothelial cells to vascularize. Hence, the MS‐containing bioink could be applied to construct 3D immunotherapeutic multicellular system for efficient skin regeneration. In addition, blood vessel formation is required to supply skin cells with adequate oxygen, nutrients, and signaling factors during the process of wound healing.^[^ ^135^ ^]^ However, insufficient vascularization may lead to infection and partial necrosi in full‐thickness defects and chronic wounds, resulting in the failure of skin regeneration.^[^ ^136^ ^]^ To address this issue, a 3D bioprinting strontium silicate (SS)‐containing multicellular scaffold was developed to serve as a vascularized skin substitute by our group.^[^ ^137^ ^]^ The SS microparticles incorporated in the hydrogel bioink served as biodegradable vascularization‐induced factors to stimulate the encapsulated fibroblast and endothelial cells by releasing bioactive ions continuously. As a result, the SS‐containing multicellular system exhibited significant angiogenesis activity both in vitro and in vivo, which inspired by the advantages of Sr and Si elements. Therefore, the SS‐containing bioengineered constructs were determined to be potential candidates for vascularized skin regeneration both in acute and diabetic wounds (Figure 7).

### 4.3 Carbon nanoparticles‐based cellular system

Carbon nanomaterials with low cytotoxicity are also allowed to be added in living systems loading with cells. For example, Bankoti et al. reported a CDs‐decorated hydrogel encapsulating stem cells to serve as a cell delivery vehicle for management of chronic wounds.^[^ ^138^ ^]^ Taking advantage of ROS scavenging ability of CDs, the antioxidant‐rich composite wound dressings had a positive effect on the healing kinetics. Simultaneously, the delivery of stem cells to wound beds significantly contributed to collagen formation and complete re‐epithelialization with reduced scarring in the animal model.

Due to the strict requirements of preparation technology and culture condition, the development of inorganic particles‐based cellular system for tissue regeneration is still in its infancy. So far, the available cellular constructs containing inorganic biomaterials for skin regeneration are limited both in type and quantity. In the construct, the inorganic particles are in direct contact with living cells for a period of time. In order to maintain the activity of the cellular system, the incorporated inorganic materials should hold low cytotoxicity with the strictly controlled dosage. In addition, when building a multicellular system, the sensitivity of different types of cells to the inorganic biomaterials should also be considered in an integrated manner. Therefore, novel inorganic particle‐based living systems will continue to be exploited with the progress of biofabrication technology.

Representative types of the inorganic biomaterials can be applied to skin tissue engineering are shown in Table 1.

**TABLE 1:** Inorganic biomaterials for skin regeneration

Application forms for wound healing	Type	Inorganic material	Biofunctions	Ref.
Inorganic particles/fibers	Bioglass (BG)	Cu‐BG, Ag‐BG	Angiogenesis Antibacterial activity Hemostasis	^[^ ^19^, ^20^ ^]^
	Mesoporous silica nanoparticles (MSNs)	Ce‐MSN, AgCa‐MSN	Reduce ROS level Tissue adhesive Antibacterial activity Hemostasis	^[^ ^23^, ^24^ ^]^
	Metal/metal oxide nanoparticles	Ca, Au, Pd, Pt, Cu CeO_2_, Tb_4_O_7_	Angiogenesis Antibacterial activity	^[^ ^26^, ^27^, ^28^, ^29^, ^30^, ^31^ ^]^
Inorganic particles‐incorporated composites	Bioceramics Bioglass (BG)	Zn‐, Cu‐, Ag‐, Co‐, Ce‐, Eu‐BGs	Angiogenesis Antibacterial activity Anti‐inflammation Hemostasis Tissue adhesive	^[^ ^39^, ^40^, ^41^, ^44^, ^45^, ^46^, ^47^, ^48^ ^]^
	Silicates	CaSiO_3_, Ca_2_ZnSi_2_O_7_, Ca_7_P_2_Si_2_O_16_, CaCuSi_4_O_10_, copper silicate, manganese silicate	Angiogenesis Antibacterial activity Hair follicle regeneration Melanoma therapy	^[^ ^36^, ^51^, ^52^, ^54^, ^55^, ^57^, ^58^ ^]^
	Nanoclays	Halloysite, montmorillonite, laponite	Hemostasis Tissue adhesive Mechanical strength enhancement Antibacterial activity	^[^ ^61^, ^62^, ^63^, ^64^, ^66^, ^67^, ^68^, ^69^, ^70^ ^]^
	Hydroxyapatite	Ag‐HAP, Co‐HAP		^[^ ^71^, ^72^, ^74^, ^75^ ^]^
	Metal nanoparticles	AuNPs	Antibacterial activity Angiogenesis	^[^ ^82^, ^83^, ^84^ ^]^
		AgNPs		^[^ ^86^, ^87^, ^88^ ^]^
		CuNPs		^[^ ^90^, ^91^ ^]^
		SeNPs		^[^ ^92^ ^]^
	Metal oxide nanoparticles	ZnO	Antibacterial activity Anti‐inflammation Antioxidant capacity Angiogenesis	^[^ ^94^, ^95^, ^96^ ^]^
		TiO_2_		^[^ ^97^, ^98^, ^99^ ^]^
		CeO_2_		^[^ ^101^, ^102^ ^]^
		CuO		^[^ ^124^, ^139^ ^]^
		Iron oxides		^[^ ^105^ ^]^
		Terbium hydroxide		^[^ ^103^ ^]^
	Carbon nanomaterials	Graphene, graphene oxide	Mechanical reinforcement Antibacterial activity Tissue adhesive	^[^ ^109^, ^110^ ^]^
		Carbon nanotubes (CNTs)		^[^ ^111^, ^112^ ^]^
		Carbon dots (CDs)	Antibacterial activity Anti‐inflammation pH sensitivity	^[^ ^116^, ^117^ ^]^
		Nano diamonds (NDs)	Hydrophilicity Biosensing	^[^ ^119^, ^120^ ^]^
	Other	CuS	Hemostasis	^[^ ^121^ ^]^
		Antimonene nanosheets	Antibacterial activity	^[^ ^122^ ^]^
		Ti3C2Tx MXene	Antibacterial activity Reduce cytotoxicity of AgNPs	^[^ ^125^ ^]^
Inorganic particles‐based living cellular systems	BG + cells	BG	Angiogenesis Collagen deposition	^[^ ^129^, ^130^ ^]^
	Silicates + cells	manganese silicate	Immunoregulation	^[^ ^133^ ^]^
		strontium silicate	Angiogenesis	^[^ ^137^ ^]^
	Carbon nanomaterial + cells	Carbon dots (CDs)	Antioxidant capacity	^[^ ^138^ ^]^

## 5 PREPARATION METHODS OF INORGANIC PARTICLE‐BASED COMPOSITE BIOMATERIALS

### 5.1 Traditional methods

A common method for fabricating inorganic particles‐based composite biomaterials is to simply mix inorganic components with the polymers by ultrasonic energy or heating.^[^ ^140^ ^]^ Then, the mixed solution can be placed in a mold to cross‐link and cured to cover the skin injury, or be directly injected to the wound site for in situ deposition.^[^ ^110^, ^141^ ^]^ Therefore, these traditional approaches have been widely used in the past for a long time due to convenient preparation procedures and wide applicability.^[^ ^142^ ^]^

Moreover, the methods of integrating inorganic particles and polymers in a non‐blending mode are also adopted in previous studies. For instance, Bao et al. prepared a multi‐layered composite wound dressing by hot pressing a modified polymer membrane with a BG layer.^[^ ^143^ ^]^ The results showed that the modified polymer layer played a key role in absorbing exudates and transferring bioactive ions from BG layer to the wound bed. Therefore, the purpose of connecting inorganic materials and polymers can also be achieved by applying external forces (hot pressing, molding, vacuum filtration, spraying, etc.) to fabricate composite films/membranes with a hierarchical structure for wound healing.^[^ ^52^, ^109^, ^144^ ^]^

### 5.2 Electrospinning

Electrospinning has become a reliable technology to prepare tissue engineering scaffolds because it can fabricate biomimetic systems similar to the morphology of natural extracellular matrix (ECM).^[^ ^145^ ^]^ From the perspective of wound healing, the electrospun wound dressings can meet main requirements outlined for wound management such as hemostasis, exudate absorption, antibacterial, and gaseous exchange, due to their polyporous and microfibrous structure.^[^ ^145b^ ^]^ With the aim of enhancing bioactivities, the methods of incorporating inorganic particles into polymer solution to obtain composite micro/nanofibers have been extended to make a mixed solution of organic/inorganic biomaterials for electrospinning. The concentrations, molecular weights, viscosity, surface tension, conductivity/surface charge density of the mixed solution significantly affect the morphology and properties of composite nanofibers.^[^ ^146^ ^]^ Of course, inorganic particles should be incorporated into the solution for electrospinning at an appropriate content to ensure the successful preparation of nanofibers and enhance the biological functions of the nanofibrous membranes.

### 5.3 Three‐dimensional (3D) printing

In recent years, great progress has been made in skin tissue engineering using 3D printing technology. During the procedure of 3D printing, the precise spatial distribution of materials, biomolecules, and even living cells can be achieved through the layer‐by‐layer deposition.^[^ ^147^ ^]^ The potential of 3D printing technology to construct sophisticated porous architectures can make it to be widely applied to the cutaneous wound therapies and skin tissue reconstruction. Therefore, the open and porous 3D constructs can be fabricated with any desired micropattern to satisfy the needs of clinical application. With the aim to fabricate bioactive scaffolds, researchers blend inorganic biomaterials into polymers to develop composite inks for printing, leading to specific physical, chemical, and biological performances of the composite scaffolds. As reported by Siebert, the 3D printed t‐ZnO‐laden composite patches showed uniform distribution and sufficient contact area of ZnO nanoparticles inside the hydrogel, leading to enhanced structural stability and homogeneous diffusion of bioactive factors.^[^ ^95^ ^]^ Besides, Wu and Hong reported a novel AgNP‐crosslinked nanocomposite hydrogel.^[^ ^148^ ^]^ Combining silver‐ethylene interaction and 3D printing technique, the nanocomposite platform held high water uptake rate and antibacterial activity. As a result, the AgNP‐containing composite dressing could accelerate wound healing and restrain scar formation.

### 5.4 Bioprinting

On the basis of 3D printing, the bioprinting technology has emerged as an advanced method for the fabrication of complex living tissues and organs.^[^ ^149^ ^]^ Through 3D bioprinting, the controllable spatial distribution of inorganic biomaterials and living cells enables specific material‐cell and cell‐cell interactions to be realized.^[^ ^149^, ^150^ ^]^ While still in its infancy, bioprinting strategies have demonstrated their enormous potential in construction of composite skin tissue‐engineered scaffolds.^[^ ^1^, ^141^, ^151^ ^]^ According to the recent studies of our group, the significantly positive effects of the incorporation of bioactive silicates into bioinks for 3D bioprinting multicellular skin substitutes has been investigated.^[^ ^133^, ^137^ ^]^ It should be emphasized that the bioactive inorganic/organic composite bioinks containing silicate‐based micro/nanoparticles have the dual functions of loading cells and stimulating cell differentiation for tissue regeneration. During the bioprinting process, the two modes, including direct printing of the cell‐encapsulated bioink incorporating with inorganic particles and alternate printing of the inorganic/organic composite ink and living cells layer by layer, can be adopted to establish the engineered living system for skin regeneration.

**FIGURE 8:**
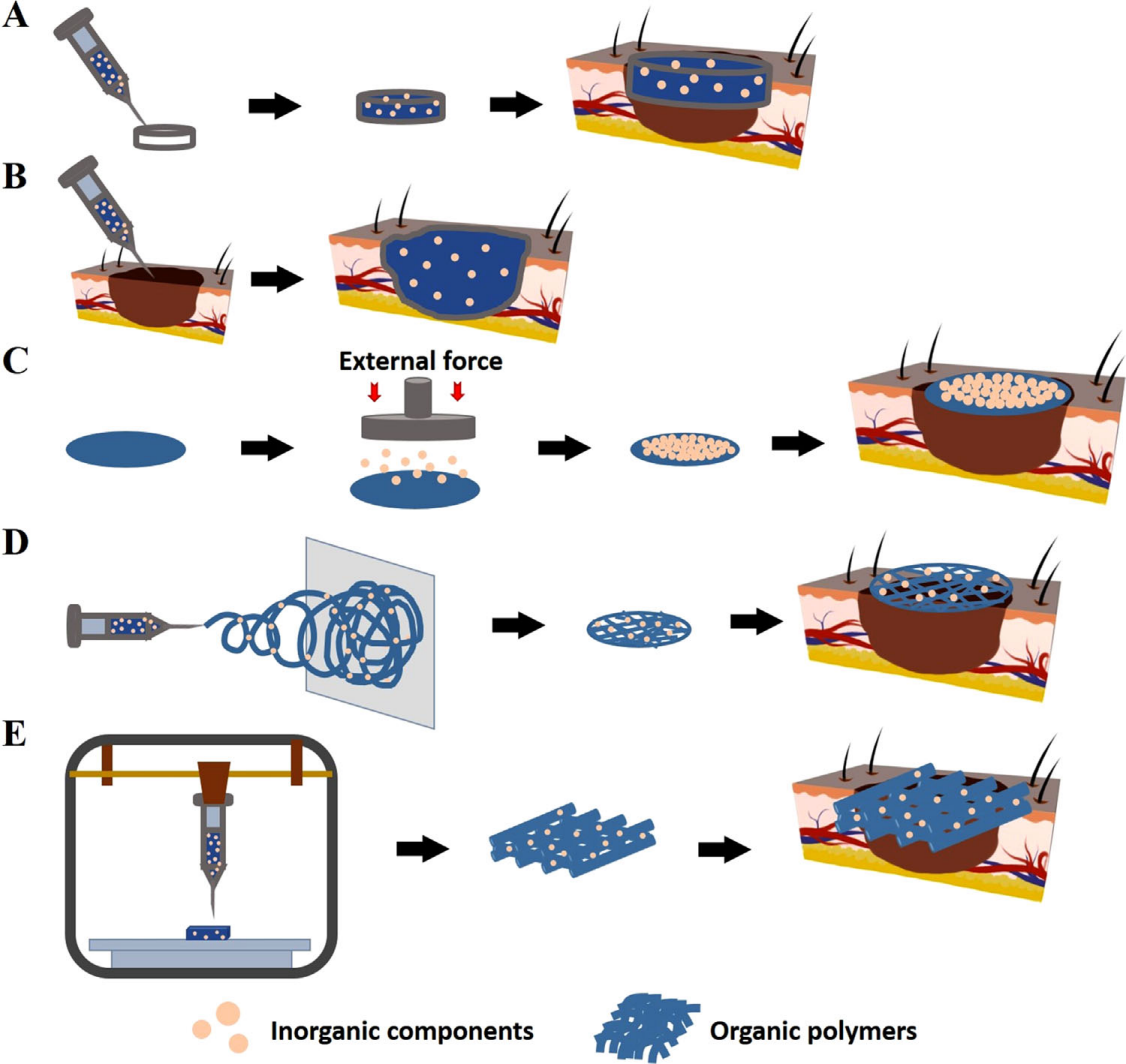
Schematic illustration for approaches to establish inorganic particles‐based composite biomaterials for wound healing. Preparation methods of (A) pre‐gelation, (B) in situ deposition, (C) non‐blending mode, (D) electrospinning, and (E) 3D printing can be adopted to produce diverse forms of skin tissue‐engineering scaffolds

Various forms of inorganic particles‐based composite biomaterials prepared by different methods for treatment of cutaneous wound are shown in Figure 8. The main types of scaffolds used for wound healing are listed in Table 2.

**TABLE 2:** Main types of the scaffold for skin tissue engineering

Preparation methods	Types of scaffold	Micropattern
Traditional methods		
Blending mode	Bulk	No
Non‐blending mode	Multi‐layered scaffold	No
Electrospinning	Microfibrous membrane	Yes
Three‐dimensional (3D) printing	Layered porous scaffold	Yes
Bioprinting	Layered porous multicellular scaffold	Yes

## 6 CONCLUSION

In summary, the development of inorganic particles‐based biomaterials for skin tissue engineering has aroused widely attention. In fact, with the rapid development of advanced technology, a variety of novel inorganic biomaterials have been designed for wound healing during the last few years. The application of inorganic particles in the process of skin tissue regeneration has undergone three stages of evolution, including the direct use of inorganic materials, inorganic‐organic composite biomaterials, and inorganic particles‐based living cellular systems. So far, the incorporation of specific inorganic components endows the engineered skin substitutes with practical functions, including hemostasis, angiogenesis activity, immunoregulation, antibacterial activity, promoting hair follicle regeneration, and high mechanical strength.

It is worth noting that the most prominent advantage of inorganic biomaterials is that they can greatly improve the physical, chemical, or biological properties of skin substitutes. For example, inorganic fillers can reinforce the mechanical strength of composite wound dressings, with the aim to meet the practical application needs of treating cutaneous wound well.^[^ ^71^, ^109^ ^]^ For the 3D printing of skin scaffolds, the incorporation of inorganic particles/fibers may promote the printability of inks.^[^ ^133^ ^]^ More importantly, the bioactive ions released from the inorganic particles can regulate the expression levels of gene by stimulating cell signaling pathways, thereby affecting one or more links during the wound healing process (immune response, angiogenesis, anti‐infection, etc.) and inducing new tissue formation rapidly. However, the limitation of inorganic biomaterials lies in the difference between the mechanical properties of the hard material and that of the soft skin tissue.^[^ ^15^ ^]^ The difference may adversely affect cells and surrounding tissues, reducing the efficiency of skin regeneration. Therefore, the difficulty of combining inorganic material and soft tissue hinders the application of inorganic particles in the field of skin tissue engineering to a certain extent. In addition, the degradation rate and degradation products of inorganic materials are also key factors that affect skin regeneration significantly. It can not be ignored that the mismatch between the rates of material degradation and skin tissue regeneration, and the toxicity of degradation products have become the main obstacles to the application of inorganic particles for skin repair.

In the future, the development of inorganic biomaterials in skin tissue engineering still needs to be further explored. On the one hand, more types of novel inorganic biomaterials that possess the potential to be integrated into wound dressings to exert unique biological effects should be designed and fabricated. We hypothesize that different combinations of multiple elements and ions provided by novel inorganic biomaterials will have special impacts on cell activities, and then regulate the wound healing process, directly or indirectly. Therefore, there are some new members of inorganic biomaterials that can be used to accelerate skin tissue repair that are waiting for researchers to discover. On the other hand, the optimization of the existing inorganic biomaterials cannot be ignored. Improving preparation methods, material properties, and biological activities can help the inorganic components to satisfy the application needs of wound dressings for effective skin regeneration. In particular, the promotion of the biocompatibility of inorganic particles is crucial for their application in living cellular systems. On this basis, the development direction of functional skin substitutes will gradually shift from “non‐living material” to “living material‐cell intergrant” in the field of skin tissue engineering.

However, preparation of inorganic particles‐based biomaterials with multifunctional properties still remains significant challenge. With the aim to meet diverse application requirements, future research on inorganic particles‐based composite wound dressings should be directed to design and fabricate novel “smart” material systems with multiple biofunctions.^[^ ^152^ ^]^ Especially, the tissue‐engineered skin substitutes combing bioactive inorganic biomaterials and multiple skin cells will become promising candidates for treatment of severe full‐thickness skin defects. Benefiting from the stimulation of bioactive inorganic particles, activities of the encapsulated cells are regulated, making it possible for the skin substitute to establish a highly biomimetic microenvironment to successfully integrate with the host tissue and replace injured skin permanently. During the skin repair, the regeneration of microstructures such as blood vessels, nerves, hair follicles, sweat glands, and sebaceous glands in the regenerated tissue remains the focus of future research on skin tissue engineering. In this respect, it cannot be ignored that regarding to the interactions between multi‐cells and inorganic biomaterials involved in the skin substitutes will guide the field towards material design. In addition, production cost and regulatory approval procedure are considered to be critical factors to limit the clinical availability of inorganic particles‐based biomaterials at present. It should be emphasized that successful bench‐to bedside translation is ultimate goal of material research. Therefore, the detailed performances (physical, chemical, biological properties) and long‐term toxicity of inorganic particles‐based biomaterials under in vivo conditions are indeed worthy of investigation in the future. Overall, multifunctional bioactive inorganic particles‐based biomaterials are primed to exert a lasting impact on skin regeneration for many years to come.

## CONFLICT OF INTEREST

The authors declare no conflict of interest
